# Cost–Effectiveness of Newborn Screening for X-Linked Adrenoleukodystrophy in the Netherlands: A Health-Economic Modelling Study

**DOI:** 10.3390/ijns11030053

**Published:** 2025-07-16

**Authors:** Rosalie C. Martens, Hana M. Broulikova, Marc Engelen, Stephan Kemp, Anita Boelen, Robert de Jonge, Judith E. Bosmans, Annemieke C. Heijboer

**Affiliations:** 1 Endocrine Laboratory, Department of Laboratory Medicine, Amsterdam UMC Location University of Amsterdam, 1105 AZ Amsterdam, The Netherlands; 2 Endocrine Laboratory, Department of Laboratory Medicine, Amsterdam UMC Location Vrije Universiteit Amsterdam, 1081 HV Amsterdam, The Netherlands; 3 Amsterdam Gastroenterology Endocrinology Metabolism, 1105 AZ Amsterdam, The Netherlands; 4 Department of Health Sciences, Faculty of Science, Amsterdam Public Health Research Institute, Vrije Universiteit Amsterdam, 1081 HV Amsterdam, The Netherlands; 5 Department of Paediatric Neurology, Amsterdam Leukodystrophy Centre, Emma Children’s Hospital, Amsterdam Neuroscience, Amsterdam UMC Location University of Amsterdam, 1105 AZ Amsterdam, The Netherlands; 6 Department of Laboratory Medicine, Laboratory Genetic Metabolic Diseases, Amsterdam Neuroscience, Amsterdam Gastroenterology Endocrinology Metabolism, Amsterdam UMC Location University of Amsterdam, 1105 AZ Amsterdam, The Netherlands; 7 Amsterdam Reproduction & Development Research Institute, 1105 AZ Amsterdam, The Netherlands; 8 Department of Laboratory Medicine, Amsterdam UMC Location University of Amsterdam, 1105 AZ Amsterdam, The Netherlands; 9 Department of Laboratory Medicine, Amsterdam UMC Location Vrije Universiteit Amsterdam, 1081 HV Amsterdam, The Netherlands

**Keywords:** adrenoleukodystrophy, newborn screening, cost–effectiveness, Markov model, quality-adjusted life-years

## Abstract

X-linked adrenoleukodystrophy (ALD) is an inherited metabolic disorder that can cause adrenal insufficiency and cerebral ALD (cALD) in childhood. Early detection prevents adverse health outcomes and can be achieved by newborn screening (NBS) followed by monitoring disease progression. However, monitoring is associated with high costs. This study evaluates the cost–effectiveness of NBS for ALD in The Netherlands compared to no screening using a health economic model. A decision tree combined with a Markov model was developed to estimate societal costs, including screening costs, healthcare costs, and productivity losses of parents, and health outcomes over an 18-year time horizon. Model parameters were derived from the literature and expert opinion. A probabilistic sensitivity analysis (PSA) was performed to assess uncertainty. The screening costs of detecting one ALD case by NBS was EUR 40,630. Until the age of 18 years, the total societal cost per ALD case was EUR 120,779 for screening and EUR 62,914 for no screening. Screening gained an average of 1.7 QALYs compared with no screening. This resulted in an incremental cost–effectiveness ratio (ICER) of EUR 34,084 per QALY gained for screening compared to no screening. Although the results are sensitive to uncertainty surrounding costs and effectiveness due to limited data, NBS for ALD is likely to be cost–effective using a willingness-to-pay (WTP) threshold of EUR 50,000– EUR 80,000 per QALY gained.

## 1. Introduction

X-linked adrenoleukodystrophy (ALD) is an inborn metabolic disorder that can cause the impairment of the adrenal glands and leukodystrophy during childhood [1]. ALD is caused by pathogenic variants in the *ABCD1* gene which is located on the X-chromosome. The disease manifests differently in male and female patients. The dysfunction of *ABCD1* causes the impaired degradation of very long chain fatty acids (VLCFA; >C22:0) in peroxisomes, and VLCFA accumulation in organs [2]. At birth, affected males are asymptomatic but later in life they can develop adrenal insufficiency, leukodystrophy (cerebral ALD; cALD), and/or spinal cord disease (adrenomyeloneuropathy; AMN) [3]. Adrenal insufficiency and cALD often manifest in childhood, while AMN develops in (late) adulthood [3]. There is no genotype–phenotype correlation, and clinical symptoms that will develop in an individual with ALD cannot currently be predicted [4].

Adrenal insufficiency is often the first manifestation of ALD in male children with a lifetime prevalence of about 85% and onset before the age of 10 years old in 50% [3,5]. Adrenal insufficiency results in cortisol deficiency [5]. Symptoms are initially non-specific, such as fatigue and nausea, so it is not easy to make a clinical diagnosis. If left untreated, low cortisol levels can result in an adrenal crisis and eventually death [6]. Treatment consists of cortisol replacement therapy [2,6,7].

Leukodystrophy has a lifetime prevalence of about 60%, with onset before the age of 18 years in 40%, with peak incidence around 6–8 years of age [1,8,9]. The onset of leukodystrophy can be detected on brain MRI long before the onset of symptoms. Progression can be halted by allogeneic hematopoietic stem cell transplantation (HSCT), but the outcome is only favourable in those patients that are treated in the asymptomatic stage (i.e. leukodystrophy on MRI in an early stage without symptoms) [4,10,11,12,13,14]. If cALD is detected once symptoms have started, HSCT is ineffective and patients enter a vegetive state within two to three years after the first neurologic symptoms of cALD occur [4]. This means that boys at risk receive regular MRI scans to detect the onset of leukodystrophy and initiate treatment [7].

To prevent adverse health outcomes, the early detection of adrenal failure and leukodystrophy is necessary, and can be achieved by monitoring patients with ALD from birth [7]. Without newborn screening, only boys with a family history of ALD are identified before the onset of symptoms [5]. The inclusion of ALD in the newborn screening program (NBS) enables the diagnosis and monitoring of all incident cases. Based on the Wilson and Junger criteria, for any disease to be included in the NBS, screening should result in relevant health gains, and an evidence-based screening algorithm of high quality should be available [15]. It is expected that ALD screening fulfils these criteria, since the early diagnosis and subsequent monitoring of ALD should result in gains in life expectancy and quality of life. With regard to the second criterion, a pilot study in 2021 showed that the screening algorithm for ALD, which was developed by Amsterdam UMC in collaboration with the National Institute for Public Health and the Environment (RIVM), has a high sensitivity and specificity [16,17]. Based on these results, ALD was added to the NBS program of The Netherlands on 1 October 2023 [18]. Although cost–effectiveness is not a criterion for deciding whether to add a specific screening to the Dutch NBS program, the limited healthcare budget raises the question of whether screening for rare diseases such as ALD is cost–effective. It is therefore essential to evaluate the cost–effectiveness of ALD screening in order to inform future policy, optimise resource use, and support the ongoing sustainability of the newborn screening program as it continues to expand. The monitoring of identified ALD cases allows for the early detection of adrenal insufficiency and cALD, resulting in substantial health gains, but also in high costs, as affected boys receive regular brain MRIs and ACTH tests, and HSCT when cALD is present. Therefore, the aim of this study is to evaluate the cost–effectiveness of NBS for ALD in The Netherlands by comparing screening for ALD (screening) with no screening for ALD in NBS (no screening) from a societal perspective, and over a time horizon of 18 years using a health-economic model.

## 2. Methods

### 2.1. Model Structure

A decision tree in combination with a Markov model was developed to simulate average health outcomes and costs of screening for ALD in NBS with no screening for ALD. In general, the main analysis was conducted from a societal perspective according to the Dutch guidelines for economic evaluations [19]. Health outcomes consisted of quality adjusted life years (QALYs) gained. Incremental cost–effectiveness ratios (ICER) in Euro per QALY gained were estimated. The ICER was considered cost–effective when it did not exceed the willingness-to-pay (WTP) threshold of EUR 50,000 per QALY. According to the Dutch guidelines for economic evaluations, the WTP ranges between EUR 20,000 and EUR 80,000 per QALY gained depending on the severity of the disease [19,20]. Considering the severity of ALD, we expect that the most relevant range for the WTP is EUR 50,000 to EUR 80,000 per QALY gained.

### 2.2. Decision Tree—ALD Newborn Screening

**Figure 1:**
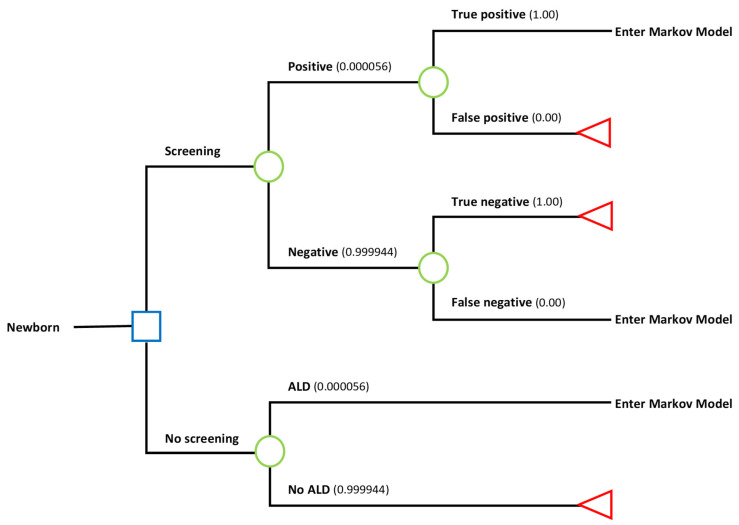
Decision tree initial NBS versus no screening with probabilities. Blue square: decision node; green circle: chance node; red triangle: outcome node.

A decision tree was developed to estimate the costs of screening for ALD in NBS per child screened (Figure 1). The screening algorithm for ALD consists of four tiers, the first tier measures the concentration of C26:0LPC [16]. The second tier is the determination of the sex of the child, using the X-counter, to select only boys [17]. The third tier also measures the concentration of C26:0LPC but with a method with a higher specificity and sensitivity and thereby a lower cut-off value than in the first tier. The fourth tier consists of the *ABCD1* gene sequencing, to identify boys with a pathogenic variant in the *ABCD1* gene. The cost of these four tiers together is EUR 2.21 per screened child (Table 1). After a positive NBS result for ALD, the child is referred by the general practitioner (GP) to the paediatric neurologist, and during the visit to the paediatric neurologist a blood sample is drawn and analysed to confirm the NBS result (EUR 1694) [16,17]. In this blood sample, the concentration of C26:0LPC is measured and *ABCD1* gene sequencing is performed (same as in tier 3 and 4 of the NBS algorithm) [17]. If the NBS result is confirmed, the result is true-positive (Figure 1), and a visit to the clinical geneticist is scheduled for extended family screening (EUR 1580). The total cost of the path of a true-positive result in the decision tree of Figure 1 includes the screening itself, confirmation of the positive result and extended family screening and is in total EUR 3276.

The probabilities in the decision tree were based on the Dutch pilot study by Albersen et al. [16]. In the pilot study, four boys with ALD were found in a cohort of 71,208 children, resulting in a probability of a positive result of 0.000056. We assumed that the probability of obtaining a false-positive or false-negative result was zero, as no false-positives or false-negatives were identified in the pilot study. Additionally, we assumed that there were no false-positives as the last tier of the screening algorithm consists of genetic screening. The probability of having ALD if not screened was assumed to be the same as the probability of having a true-positive result for ALD if screened. Approximately 170,000 children are screened in the Dutch NBS program per year. Based on the Dutch pilot study and the total number of children screened per year, we expect an average of 10 ALD cases per year ((170,000/71,208)*4).

### 2.3. Markov Model—Disease Progression

To simulate the natural course of ALD, a Markov model was developed with a time-horizon of 18 years, a cycle length of six months, and cohort size of 1000 individuals. A time-horizon of 18 years was chosen because the majority of boys with ALD will develop adrenal insufficiency and/or cALD in the first 18 years of their life [3]. A cycle length of six months was chosen, because the monitoring guidelines for ALD recommend adrenal function testing and monitoring for cerebral onset by brain MRI each six months [7].

**Figure 2:**
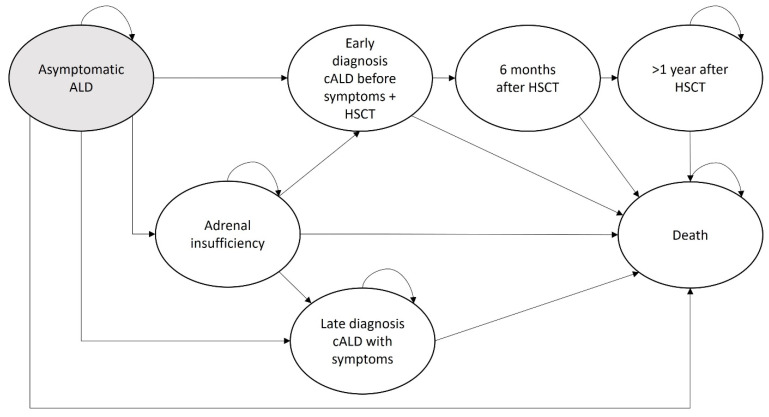
Schematic Markov model disease progression ALD. ALD adrenoleukodystrophy; cALD cerebral ALD; HSCT hematopoietic stem cell transplantation; AI adrenal insufficiency.

The model consisted of seven health states (Figure 2). All boys with an ALD mutation entered the model in the asymptomatic ALD health state, as they are asymptomatic at birth [3]. From the asymptomatic health state, affected individuals can progress to several health states. For the screening group, we assumed that the probability of progressing to late diagnosis cALD is zero because regular brain MRIs detect early leukodystrophy, after which HSCT is performed to prevent disease progression [7]. Conversely, in the no screening group, we assumed that the majority of patients who develop cALD are diagnosed with cALD only if symptoms are present, which means that treatment is no longer effective and eventually leads to death in a timeframe of two to three years after the first neurological symptoms [4]. The likelihood of progression from asymptomatic ALD to adrenal insufficiency is similar in both groups. A more detailed explanation of disease progression can be found in Supplemental S1.

### 2.4. Model Parameters

All model parameters were derived from literature or expert opinion (Table 1).

#### 2.4.1. Transition Probabilities

Age-specific transition probabilities from the asymptomatic health state to the health states adrenal insufficiency or cALD were applied, as progression differs per age group [3]. For the other health states, no age-specific transition probabilities were used since these transitions do not differ between age groups. Transition probabilities were recalculated to six-month probabilities (Table 1), the original probabilities with a description of the recalculation to six months probabilities are displayed in Supplemental S2, Table S1.

#### 2.4.2. Costs

Costs were assessed from a societal perspective and a discount rate of 3.5% was applied according to Dutch guidelines [19,21]. The following costs were included: screening costs, monitoring costs, treatment costs, care costs and productivity loss from parents (see Table 1). The monitoring costs differed per health cycle, as the monitoring guidelines change with age. To monitor for the onset of adrenal insufficiency, the guidelines recommend that ACTH needs to be measured every 6 months in boys between six months and 10 years of age and annually after 10 years of age [7]. The guidelines for monitoring cALD recommend that boys have a brain MRI under anaesthesia twice a year between the ages of 2 and 7 years, without anaesthesia twice a year between ages 7 and 12 and annually after the age of 12 years. The monitoring costs in the asymptomatic health state of the no screening group are zero, because patients do not know they have ALD and are therefore not monitored for disease progression.

Treatment costs for adrenal insufficiency consist of hydrocortisone suppletion and every 6 months a visit to the paediatric endocrinologist. The treatment costs of early cALD include the costs of the HSCT treatment and care after the HSCT treatment, which means visits to the haematologist every week until one year after HSCT. After the first year of regular check-ups after HSCT, the patient visits the paediatric neurologist once a year. The costs associated with late cALD included home care, consisting of an hour of medical care and an hour of personal care per day. Productivity losses for parents were considered during HSCT and cALD, since one of the parents often stops with paid work to take care of their child during these health states. Productivity losses were calculated by multiplying the costs per working hour from the cost manual by the average number of worked hours per week according to CBS data [21,22]. To derive the productivity losses per half a year, these average costs per week were multiplied by the number of weeks per half-year.

#### 2.4.3. Quality-Adjusted Life-Years

Effects were expressed as quality-adjusted life-years (QALYs), which were discounted using a discount rate of 1.5% [19]. Utilities for the health states related to adrenal insufficiency and early cALD were estimated by mapping the Paediatric Quality of Life Inventory (PedSQL) on the EQ-5D using an existing algorithm [24,25,26,29]. For the health state adrenal insufficiency, PedSQL scores in children with congenital adrenal hyperplasia (CAH) were used because CAH also affects the adrenal glands [24]. For the health states early cALD with HSCT and 6 months after HSCT, we used PedSQL scores from children who underwent HSCT [25,26]. According to the literature, boys with cALD who have undergone HSCT have the same quality of life at one year post transplant as healthy children, so we used the same utilities as for the health state adrenal insufficiency, because the majority of these boys have adrenal insufficiency [27]. The utilities for late cALD were based on the study of Bessey et al. in combination with expert opinion [28]. To estimate QALYs, the utilities were multiplied by the time spent in a health state.

### 2.5. Deterministic Sensitivity Analysis

A deterministic sensitivity analysis was conducted to estimate the sensitivity of the mapped utilities by using the 95% confidence interval limits around the PedSQL scores. We also performed a threshold analysis to assess the impact of changes in the probability of dying from late cALD three years post onset at 50%, 60%, 70% and 80% instead of 90%. For the reason that patients do not necessarily die from late cALD, but they eventually enter a vegetative state where quality of life is low and palliative care may be withdrawn, depending on national and personal preferences. Furthermore, we performed a sensitivity analysis in which the probability of developing cALD before the age of 18 was set to 20% instead of 35%, as the probability of developing cALD is likely to be overestimated in the literature, as based on expert opinion. In addition, we analysed the change in the ICER value if the X-counter (tier 2) is excluded from the screening algorithm, as it is possible to take the sex information from the dried blood spot card instead. The cost for the X-counter is EUR 0.59 per newborn screened. Finally, we assessed the effect of a change in the ICER when comparing a scenario in which five cases are detected in one year instead of 10 cases (based on the pilot study).

### 2.6. Probabilistic Sensitivity Analysis

To estimate uncertainty surrounding the deterministic results, a probabilistic sensitivity analysis (PSA) was performed using Monte Carlo simulation (10,000 simulations). The PSA was conducted around the transition probabilities and utilities. Beta distributions were fitted for transition probabilities using data on the natural history of ALD from Huffnagel et al. [3]. For utilities, beta distributions were also used, and the alpha and beta were estimated based on the 95% confidence interval of the mapped utilities. Table 1 shows the alpha and beta values used. Costs were considered fixed in the PSA because they were based on standard treatment protocols, reference prices, and market prices. We assumed that uncertainty was small for these types of costs. A cost–effectiveness plane (CE plane) was plotted to display the uncertainty surrounding the ICER, and a cost–effectiveness acceptability curve (CEAC) was plotted to show the probability that screening is cost–effective compared to no screening at different willingness-to-pay (WTP) thresholds.

## 3. Results

### 3.1. Screening Costs

Based on the decision tree in Figure 1, we estimated that the cost of screening for ALD in the NBS was EUR 2.39 per newborn screened (including the screening itself, the cost of confirming a positive result, and extended family screening). When expecting an average of 10 ALD cases per year from 170,000 screened children in total, the screening costs of the ALD cohort to detect one ALD case through NBS was EUR 40,630 (EUR 2.39*170,000/10) (Table 2).

### 3.2. Effectiveness

The model predicted that screening resulted in an average of 15.2 QALYs per child with ALD until the age of 18 compared to 13.5 QALYs for no screening, meaning that screening results in a gain of 1.7 QALYs compared to no screening (Table 3). Furthermore, the incremental life years of a screened child with ALD were 1.9 years compared to an unscreened child with ALD (Table S2 in Supplemental 3). The QALYs and life years for screened boys are higher because boys who are screened for ALD do not progress to the irreversible state of late cerebral ALD with symptoms.

### 3.3. Costs

The predicted costs from a societal perspective were EUR 120,779 per child with ALD in the screening group, and EUR 62,914 per child with ALD in the no screening group (Table 2 and Table 3). Thus, the incremental costs from a societal perspective were EUR 57,865 per child with ALD. Table 2 shows the costs per cost category. Monitoring costs were EUR 17,157 in the screening cohort and EUR 1769 in the no screening cohort, as boys with a pathogenic *ABCD1* variant in the screening cohort are monitored from birth for their disease progression. Also, ALD treatment costs were higher in the screening cohort (EUR 46,524) compared to the no screening cohort (EUR 38,346), which is explained by the fact that boys in the screening cohort more often receive HSCT for early cALD, which is an expensive treatment. The productivity losses of the parents represent a relatively minor component of the total costs. In the screening group, productivity losses were EUR 16,468, as the parents of boys undergoing HSCT experience productivity losses due to the need to care for their child and frequent hospital visits during HSCT. In the no screening cohort, the productivity losses were higher: EUR 22,799. The parents of boys who progress to late cALD have higher productivity losses because one of the parents usually stops working during this period to care for their child.

### 3.4. Cost–Effectiveness

The ICER of the deterministic cost–effectiveness analysis per child with ALD until the age of 18 years was EUR 34,084 per QALY gained for screening compared to no screening (Table 3). The ICER per life year gained was EUR 30,649 per life year gained (Table S2 in Supplemental S3).

### 3.5. Deterministic Sensitivity Analysis

**Figure 3:**
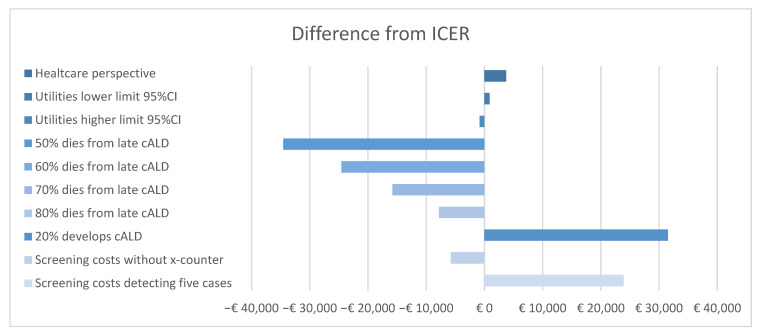
Difference from ICER in the main analysis.

The results of the deterministic uncertainty analysis are presented in Table 3 and in the tornado plot in Figure 3. From a healthcare perspective, the ICER increases to EUR 37,456 per QALY gained. The difference in costs between the groups increased, because the higher healthcare costs in the screened cohort are not offset by the lower lost productivity costs in the screened cohort as compared to the unscreened cohort (Table 2). The ICERs in the analyses using the lower and upper bounds of the 95% CI of the mapped PedSQL scores show a small difference compared to the ICER of the deterministic analysis, EUR 34,991 and EUR 33,273 per QALY gained, respectively. When the probability of dying from late cALD is changed from 90% to 50% in 3 years post onset, screening for ALD is dominant over no screening (the ICER changes to −EUR 480) due to the higher costs of home care and productivity losses because boys with cALD live longer in a vegetative state. A threshold analysis shows that screening for ALD is dominant over no screening when the probability of dying from late cALD in 3 years is less than 60%. Furthermore, reducing the probability of developing cALD before the age of 18 years to 20% increases the ICER per child with ALD to EUR 65,626 per QALY. Upon the removal of the X-counter (tier 2), the cost of detecting one case changes to EUR 30,770 and the ICER changes to EUR 28,310, as the total cost for screening decreases. In the final deterministic sensitivity analysis, we calculated the screening costs when only 5 cases were detected with screening annually instead of 10 cases. This increases the costs of detecting one case to EUR 81,260 and the ICER to EUR 58,015. Information on the ICER per life year gained from these deterministic sensitivity analyses can be found in Table S2 in Supplemental S3.

### 3.6. Probabilistic Sensitivity Analysis

**Figure 4:**
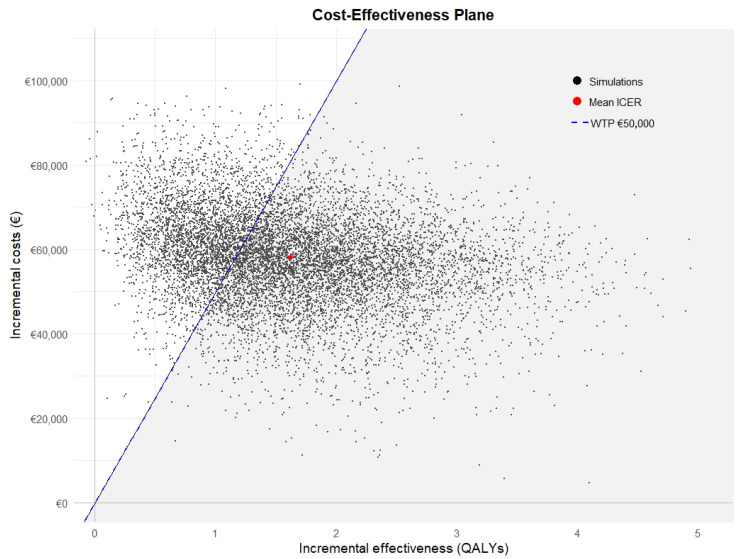
Cost–effectiveness plane of screened versus unscreened.

**Figure 5:**
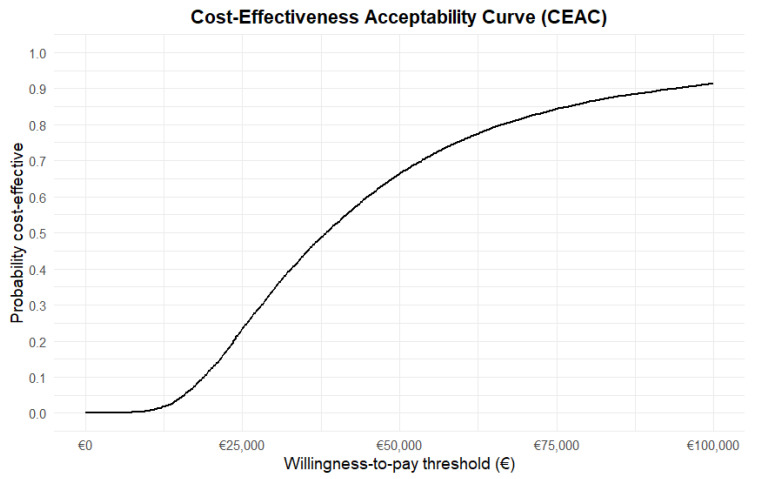
Cost–effectiveness acceptability curve of different WTP thresholds.

The cost–effectiveness (CE) plane (Figure 4) shows the 10,000 Monte Carlo simulations of the ICER for ALD screening compared to no ALD screening. The CE plane demonstrates considerable uncertainty surrounding the cost and effectiveness. However, all simulations are in the northeast quadrant, indicating that we can be reasonably certain that screening is more effective and the costs higher than not screening. The cost–effectiveness acceptability (CEAC) curve (Figure 5) shows that the probability of screening for ALD being cost–effective compared to no screening is 0.15 at a WTP of EUR 20,000 per QALY gained and 0.65 at a WTP of EUR 50,000 per QALY gained. At a WTP of EUR 80,000 per QALY gained, the probability of cost–effectiveness was 0.85.

## 4. Discussion

This economic evaluation assesses the cost–effectiveness of including ALD in the Dutch NBS program from a societal perspective for the first 18 years of life. The incremental costs associated with screening were EUR 57,865 per child with ALD and the incremental QALYs were 1.7 QALY per child with ALD. This resulted in an ICER of EUR 34,084 per QALY gained from a societal perspective. From the healthcare perspective, the ICER increased slightly compared to the societal perspective. Deterministic sensitivity analyses showed that the most important parameters were the number of cases detected annually, the development of late cALD, and death from late cALD. The results of the PSA show considerable uncertainty. The probability of screening being cost–effective at a WTP of EUR 50,000 is 0.66 and at a WTP of EUR 80,000 is 0.85.

Bessey et al. also compared the cost–effectiveness of screening versus no screening for ALD in NBS and showed that costs in the screening cohort were lower than in the no screening cohort [28]. This is in contrast to our study, where costs were higher in the screening group than in the no screening group. However, it is difficult to make a direct comparison between the results of the study by Bessey et al. First, the authors primarily focused on the progression of cALD whereas we focused on both the progression of adrenal insufficiency and cALD. Second, they estimated costs and effects using a decision tree for screening and disease progression, whereas our study used a decision tree for screening combined with a Markov model for disease progression. Furthermore, the cost perspective is different, the study by Bessey et al. used an NHS and personal social services perspective (which includes costs related to social care but is not as extensive as a societal perspective) and we used a societal perspective. Finally, the time horizon of the model is different; Bessy et al. used a lifetime horizon and we used an 18-year time horizon. Apart from the study by Bessey et al., we did not find any other studies in the literature on the cost–effectiveness of including ALD in the NBS program.

A probabilistic sensitivity analysis was performed to assess the uncertainty surrounding the cost–effectiveness of screening for ALD, which is a strength of our study. Another strength is the use of empirical data from the pilot study by Albersen et al. to estimate the diagnostic performance of the ALD screening algorithm included in the Dutch NBS [16]. This is the first economic evaluation that evaluates the cost–effectiveness of screening for ALD in the NBS program compared with no screening in The Netherlands. Although the model is based on the Dutch situation, we expect the results of the model to be generalisable to countries with similar health care characteristics as The Netherlands.

This study also has limitations. Due to the rarity of ALD, limited data was available in the literature. The estimation of accurate transition probabilities for the screening group was challenging due to the recent implementation of ALD in NBS programs. Therefore, we estimated transition probabilities based on the natural history study by Huffnagel et al. in combination with expert opinion, which downsizes the benefits of screening, especially for adrenal insufficiency [3]. Only one study with follow-up data after the implementation of ALD in NBS is available in the literature and the findings demonstrate that ALD cases identified through NBS receive an adrenal insufficiency diagnosis substantially earlier than ALD cases identified clinically [30]. Second, because of limited data, no EQ-5D scores for this specific patient population with adrenal insufficiency or cALD were available in the literature. Therefore, we estimated the utilities of adrenal insufficiency and cALD by mapping the PedSQL scores onto the EQ-5D. However, this raises concerns about the conceptual validity of the estimated utilities, which is referred to as statistical alchemy in the literature [31]. Thirdly, due to the limited availability of data, the model only included parental paid productivity losses as costs from a societal perspective. Data on the costs of unpaid work, education costs, and the impact on the quality of life of parents with a child with ALD were unavailable in the literature and were therefore excluded from the cost analysis. Lastly, the ICER is subject to variation depending on the number of cases identified during screening. An increase in the number of cases identified during screening results in a decrease in the ICER, due to the costs of identifying a single case being reduced when more cases are identified during screening. In view of the limited availability of data in the literature on several estimates included in our model and the recent implementation of ALD in NBS, it is recommended that future research re-evaluates the Dutch ALD NBS screening several years after the start of screening in order to verify the estimates of our model.

An 18-year time horizon was chosen for this economic evaluation because the majority of ALD events occur before the age of 18. However, this means that we are not able to account for potential false negatives in the NBS. For this, a lifetime horizon would be needed, as boys with ALD may develop adrenomyeloneuropathy (AMN) in adulthood. In addition, we expect the ICER of screening compared to no screening to be lower if a lifetime horizon is considered for two reasons. First, the QALYs for the screened group would be higher, because boys diagnosed with cALD before the onset of symptoms and who are successfully treated with HSCT have the same life expectancy as healthy boys. Furthermore, when the productivity losses of boys with ALD in later life are included, the cost of productivity losses would be lower in the screening group compared to the no screening group, as boys who are screened and successfully treated at a young age would be able to participate in paid employment after the age of 18.

The costs of the diagnostic pathway for unscreened boys who are diagnosed with ALD because of symptoms, are not included in this economic evaluation. We expect this to result in a lower ICER of screening compared to no screening, as healthcare costs would increase in the unscreened group. In addition, costs in the unscreened group would be higher if we include the costs of children who are diagnosed with ALD based on family history and followed intensively from birth for disease progression.

With newborn screening, the likelihood of finding a variant of uncertain significance (VUS) in the *ABCD1* gene increases by genetic screening in the last tier [32]. We did not include in our model the probability of finding a VUS. However, it is important to be aware of the potential consequences of the identification of a VUS on quality of life and associated costs. For many VUS findings, the actual clinical implications and thus the associated costs are unclear. If a substantial proportion of the detected ALD cases in reality is a VUS without clinical complications, high unnecessary costs are made related to the monitoring of ALD.

## 5. Conclusions

The inclusion of ALD in the Dutch NBS program is associated with increased QALYs and increased costs compared to no screening, resulting in an ICER of EUR 34,084. The ICER of EUR 34,084 is below the commonly used WTP threshold of EUR 50,000 per QALY gained. The PSA shows that the probability of screening being cost–effective at a WTP of EUR 50,000 is 0.66 and at a WTP of EUR 80,000 0.85. Thus, for the relevant range of WTP in The Netherlands, including ALD in the Dutch NBS program is likely to be cost–effective compared to no screening for ALD. However, the results are sensitive to the number of ALD cases identified in the screening.

## Figures and Tables

**Table 1:** Model input parameters base case analysis and PSA.

Parameter	Values	Distribution for PSA	Reference
*Six-month transition probabilities*			
*Screening*			
*Age 0–4 years*			
Asymptomatic to asymptomatic	0.9846		1-rest ^a^
Asymptomatic to adrenal insufficiency	0.0132	Beta (α = 16, β = 143)	Huffnagel et al. 2019 [3]
Asymptomatic to early cALD	0.0000	Beta (α = 0, β = 151)	Huffnagel et al. 2019 [3]
Asymptomatic to late cALD	0.0000	Beta (α = 0, β = 151)	Huffnagel et al. 2019 [3]
Asymptomatic to death	0.0022	Fixed	Under-five mortality rate WHO
*Age 4–18 years*			
Asymptomatic to asymptomatic	0.9620		1-rest ^a^
Asymptomatic to adrenal insufficiency	0.0214	Beta (α = 67, β = 92)	Huffnagel et al. 2019 [3]
Asymptomatic to early cALD	0.0164	Beta (α = 52, β = 99)	Huffnagel et al. 2019 [3]
Asymptomatic to late cALD	0.0000	Beta (α = 0, β = 151)	Huffnagel et al. 2019 [3]
Asymptomatic to death	0.0002	Fixed	Mortality rate age 5 to 9 and 10 to 14 years WHO
*Age 0–18 years*			
Adrenal insufficiency to adrenal insufficiency	0.9809		1-rest ^a^
Adrenal insufficiency to early cALD	0.0189	Beta (α = 46, β = 65)	Huffnagel et al. 2019 [3]
Adrenal insufficiency to late cALD	0.0000	Beta (α = 0, β = 111)	Huffnagel et al. 2019 [3]
Adrenal insufficiency to death	0.0002	Fixed	Mortality rate age 5 to 9 and 10 to 14 years WHO
*No Screening*			
*Age 0–4 years*			
Asymptomatic to asymptomatic	0.9846		1-rest ^a^
Asymptomatic to adrenal insufficiency	0.0132	Beta (α = 16, β = 143)	Huffnagel et al. 2019 [3]
Asymptomatic to early cALD	0.0000	Beta (α = 0, β = 151)	Huffnagel et al. 2019 [3]
Asymptomatic to late cALD	0.0000	Beta (α = 0, β = 151)	Huffnagel et al. 2019 [3]
Asymptomatic to death	0.0022	Fixed	Under-five mortality rate WHO
*Age 4–10 years*			
Asymptomatic to asymptomatic	0.9620		1-rest ^a^
Asymptomatic to adrenal insufficiency	0.0214	Beta (α = 67, β = 92)	Huffnagel et al. 2019 [3]
Asymptomatic to early cALD	0.0000	Beta (α = 0, β = 151)	Huffnagel et al. 2019 [3]
Asymptomatic to late cALD	0.0164	Beta (α = 52, β = 99)	Huffnagel et al. 2019 [3]
Asymptomatic to death	0.0002	Fixed	Mortality rate age 5 to 9 and 10 to 14 years WHO
*Age 0–18 years*			
Adrenal insufficiency to adrenal insufficiency	0.9832		1-rest ^a^
Adrenal insufficiency to early cALD	0.0083	Beta (α = 23, β = 88)	Huffnagel et al. 2019 [3]; Expert opinion
Adrenal insufficiency to late cALD	0.0083	Beta (α = 23, β = 88)	Huffnagel et al. 2019 [3]; Expert opinion
Adrenal insufficiency to death	0.0002	Fixed	Mortality rate age 5 to 9 and 10 to 14 years WHO
*Same for screening and no screening*			
*Age 0–18 years*			
Early cALD to 6 months after HSCT	0.9623		1-rest ^a^
Early cALD to death	0.0377	Beta (α = 2, β = 25)	Raymond et al. 2019 [11]
6 months after HSCT to >1 year after HSCT	0.9623		1-rest ^a^
6 months after HSCT to death	0.0377	Beta (α = 2, β = 25)	Raymond et al. 2019 [11]
>1 year after HSCT to >1 year after HSCT	0.9998		1-rest ^a^
>1 year after HSCT to death	0.0002	Fixed	Raymond et al. 2019 [11]
Late cALD to late cALD	0.6813	Fixed	Expert opinion
Late cALD to death	0.3187	Fixed	Expert opinion
***Cost parameters***			
*Screening costs initial ALD NBS*			
Tier 1 t/m 4 per screened child	EUR 2.21	Fixed	Market price
Positive screening result ^b^	EUR 1694	Fixed	Reference prices 2022 [21]
Extended family screening	EUR 1580	Fixed	Market price
*Monitoring costs*			
ACTH test + daycare	EUR 410	Fixed	Market price
Brain MRI with anaesthesia	EUR 589	Fixed	Reference prices 2022 [21]
Brain MRI without anaesthesia	EUR 254	Fixed	Reference prices 2022 [21]
Visit paediatrician	EUR 120	Fixed	Reference prices 2022 [21]
*Treatment costs*			
Hydrocortisone treatment 6 months	EUR 1427	Fixed	Market price
HSCT: HLA characterisation + HSCT	EUR 109,490	Fixed	Market price
Care first year after HSCT	EUR 10,355	Fixed	Market price
Home care late cALD 6 months	EUR 36,280	Fixed	Reference prices 2022 [21]
Visit paediatrician	EUR 120	Fixed	Reference prices 2022 [21]
*Societal costs*			
Productivity losses parents HSCT 6 months	EUR 30,540	Fixed	Reference prices 2022 [21]; CBS [22]
Productivity losses parents late cALD 6 months	EUR 30,540	Fixed	Reference prices 2022 [21]; CBS [22]
***Utility values***			
Asymptomatic	1	Fixed	
Adrenal insufficiency	0.9	Beta (α = 19.3, β = 2.1)	Lawrence et al. 2023 [23,24]
Early diagnosis cALD and HSCT	0.85	Beta (α = 27.5, β = 4.9)	Feichtl et al. 2010 [25,26]
6 months after HSCT	0.85	Beta (α = 27.5, β = 4.9	Feichtl et al. 2010 [25,26]
>1 year after HSCT	0.9	Beta (α = 19.3, β = 2.1)	Lawrence et al. 2023 [24,27]
Late diagnosis cALD	0.07	Fixed	Bessey et al. 2018 [28]; Expert opinion
Death	0	Fixed	

PSA probabilistic sensitivity analysis; cALD cerebral ALD; HSCT hematopoietic stem cell transplantation; NBS newborn screening; ACTH adrenocorticotropic hormone; ^a^ Rest means the rest of the transition probabilities of that health state; ^b^ Consisting of visit from GP > 20 min, first visit to paediatric neurologist and blood sample to confirm NBS result during visit paediatrician.

**Table 2:** Type of costs per case identified with ALD, screened versus not screened.

Type of Costs	Screened	Unscreened
Costs screening per identified case	EUR 40,630	EUR -
Costs monitoring	EUR 17,157	EUR 1769
Costs treatment	EUR 46,524	EUR 38,346
Costs productivity losses	EUR 16,468	EUR 22,799
*Total societal costs*	*EUR 120,779*	*EUR 62,914*
*Total healthcare costs*	*EUR 104,311*	*EUR 40,115*

**Table 3:** Deterministic cost–effectiveness outcomes per case identified with ALD for the first 18 years.

Analysis	Strategy	Costs	QALYs	Incr Costs	Incr QALYs	ICER
Main analysis (Societal perspective)	Screened	EUR 120,779	15.2	EUR 57,865	1.7	EUR 34,084
	Unscreened	EUR 62,914	13.5			
Healthcare perspective	Screened	EUR 104,311	15.2	EUR 64,196	1.7	EUR 37,813
	Unscreened	EUR 40,115	13.5			
Utilities lower limit 95%CI	Screened	EUR 120,779	15.1	EUR 57,865	1.7	EUR 34,991
	Unscreened	EUR 62,914	13.5			
Utilities higher limit 95%CI	Screened	EUR 120,779	15.4	EUR 57,865	1.7	EUR 33,273
	Unscreened	EUR 62,914	13.6			
50% die in three years from late cALD	Screened	EUR 120,779	15.2	−EUR 768	1.6	−EUR 480
	Unscreened	EUR 121,547	13.6			
60% die in three years from late cALD	Screened	EUR 120,779	15.2	EUR 15,888	1.7	EUR 9504
	Unscreened	EUR 104,892	13.6			
70% die in three years from late cALD	Screened	EUR 120,779	15.2	EUR 30,731	1.7	EUR 18,282
	Unscreened	EUR 90,048	13.6			
80% die in three years from late cALD	Screened	EUR 120,779	15.2	EUR 44,363	1.7	EUR 26,260
	Unscreened	EUR 76,416	13.6			
20% progressing to cALD	Screened	EUR 96,208	15.4	EUR 57,219	0.9	EUR 65,626
	Unscreened	EUR 38,988	14.5			
Screening costs without x-counter (tier 2)	Screened	EUR 110,976	15.2	EUR 48,062	1.7	EUR 28,310
	Unscreened	EUR 62,914	13.5			
Screening costs detecting five cases	Screened	EUR 161,409	15.2	EUR 98,495	1.7	EUR 58,015
	Unscreened	EUR 62,914	13.5			

Costs are presented per patient. Incr incremental; QALY quality-adjusted life-year; CI confidence interval; cALD cerebral ALD.

## Data Availability

Data archiving is not mandated but data will be made available upon reasonable request.

## Supplementary Materials

The following supporting information can be downloaded at: https://www.mdpi.com/article/10.3390/ijns11030053/s1, Table S1: Overall probabilities and time period; Table S2: Results life years gained per case identified with ALD for the first 18 years.
