# Supplementary material for: Cost–Effectiveness of Newborn Screening for X-Linked Adrenoleukodystrophy in the Netherlands: A Health-Economic Modelling Study
Source: Int J Neonatal Screen. 2025 Jul 16;11(3):53. doi: 10.3390/ijns11030053 (PMC12286135; doi:10.3390/ijns11030053)
Supplement: Supplementary file 1 [file IJNS-11-00053-s001.zip › IJNS-3556203-supplementary.pdf]

Supplemental 1: Description disease progression Markov model

All boys diagnosed with ALD entered the Markov model in the first health state; asymptomatic ALD, because they are asymptomatic at birth (1). Disease progression differs between patients in the screening and no screening groups. For boys diagnosed with ALD at birth by NBS, we assume that they can remain asymptomatic or progress from asymptomatic to either adrenal insufficiency, or early diagnosis of cALD, and that the probability of progressing to late diagnosis of cALD is zero, because they are monitored for disease progression and cALD is diagnosed on the basis of an abnormal brain MRI and treatment can be started before symptoms occur (2). In the first four cycles of the model, patients will not progress to cALD, as cALD disease progression does not manifest before the age of four and brain MRI does not show abnormalities (3). The mean age of onset is 7-8 years (4, 5)

If a boy progresses to early diagnosis of cALD he will receive HSCT treatment within a month. After HSCT, patients need to stay in the hospital for two weeks. After these two weeks, patients can go home, but they have to visit the hospital regularly. Recovery from HSCT takes about six months to a year. In the model, patients can progress from the early diagnosis of cALD + HSCT to either 6 months after HSCT or they can die from HSCT treatment. From the health state >1 year after HSCT, the patient can remain in this health state, or he can die.

The majority of patients progress to adrenal insufficiency(1, 6). They may transition to the adrenal insufficiency health state after the first cycle, as manifestation of adrenal insufficiency can begin as early as five months of age(6). The patient can either remain in this health state or additionally develop early diagnosis cALD and then progress to early diagnosis cALD + HSCT health state. This health state is a tunnel state, and patients will only remain in this health state for one cycle. After this cycle, they can either transition to 6 months after HSCT or die. From 6 months after HSCT, patients can progress to either >1 year after HSCT or death. From >1 year post-HSCT, patients can either remain in this health state or die.

Disease progression for patients who are diagnosed based on clinical symptoms differs from boys diagnosed by NBS as mentioned before. We assume that patients who are clinically diagnosed will progress to the late diagnosis of cALD if they develop cALD. Symptoms of cALD include: behavioural changes, hyperactive behaviour, difficulties in maintaining schoolwork, and attention deficits(5, 7). Without treatment, cALD leads to a vegetative state and death within three years of the onset of the first neurologic symptoms(8).

The main difference between the group diagnosed after NBS or diagnosed clinically is the probability of early diagnosis of cALD and late diagnosis of cALD. We assume that the patients diagnosed after NBS have a zero probability of progressing to late diagnosis cALD. For the group diagnosed clinically, it is the other way around; the probability of progressing to early stage cALD is zero. This means that there is a major health gain for patients diagnosed after NBS.

Supplemental 2: Calculation of six months transition probabilities

The probabilities in the parameter table in the main text were recalculated to six months transition probabilities. Firstly, the overall probability was recalculated to a one year rate with the following equation: $r = -(\ln(1-P)/t$. Then the one year rate was recalculated to a six months probability with the following equation: $p_{six\ months} = 1 - exp^{(-r*0.5)}$. Below a table with the overall probabilities (P) and the time period (t) used in the calculations.

Table S1: Overall probabilities and time period

Parameter	Probability (P)	Time period in years (t)	Reference
Screening			
Age 0-4 years			
Asymptomatic to asymptomatic	1- rest[a]		
Asymptomatic to adrenal insufficiency	0.1010	4	Huffnagel et al. 2019 (1)
Asymptomatic to early cALD	0.0000	4	Huffnagel et al. 2019 (1)
Asymptomatic to late cALD	0.0000	4	Huffnagel et al. 2019 (1)
Asymptomatic to death	0.0043	1	Under-five mortality rate WHO
Age 4-18 years			
Asymptomatic to asymptomatic	1- rest[a]		
Asymptomatic to adrenal insufficiency	0.4550	14	Huffnagel et al. 2019 (1)
Asymptomatic to early cALD	0.3710	14	Huffnagel et al. 2019 (1)
Asymptomatic to late cALD	0.0000	14	Huffnagel et al. 2019 (1)
Asymptomatic to death	0.0003	1	Mortality rate age 5 to 9 and 10 to 14 years WHO
Age 0-18 years			
Adrenal insufficiency to adrenal insufficiency	1- rest[a]		
Adrenal insufficiency to early cALD	0.4144	14	Huffnagel et al. 2019 (1)
Adrenal insufficiency to late cALD	0.0000	14	Huffnagel et al. 2019 (1)
Adrenal insufficiency to death	0.0003	1	Mortality rate age 5 to 9 and 10 to 14 years WHO
No Screening			
Age 0-4 years			
Asymptomatic to asymptomatic	1- rest[a]		
Asymptomatic to adrenal insufficiency	0.1010	4	Huffnagel et al. 2019 (1)
Asymptomatic to early cALD	0.0000	4	Huffnagel et al. 2019 (1)
Asymptomatic to late cALD	0.0000	4	Huffnagel et al. 2019 (1)
Asymptomatic to death	0.0043	1	Under-five mortality rate WHO
Age 4-10 years			
Asymptomatic to asymptomatic	1- rest[a]		

Asymptomatic to adrenal insufficiency	0.4550	14	Huffnagel et al. 2019 (1)
Asymptomatic to early cALD	0.0000	14	Huffnagel et al. 2019 (1)
Asymptomatic to late cALD	0.3710	14	Huffnagel et al. 2019 (1)
Asymptomatic to death	0.0003	1	Mortality rate age 5 to 9 and 10 to 14 years WHO
Age 0-18 years			
Adrenal insufficiency to adrenal insufficiency	1- rest[a]		
Adrenal insufficiency to early cALD	0.2072	14	Huffnagel et al. 2019 (1); Expert opinion
Adrenal insufficiency to late cALD	0.2072	14	Huffnagel et al. 2019(1); Expert opinion
Adrenal insufficiency to death	0.0003	1	Mortality rate age 5 to 9 and 10 to 14 years WHO
Same for screening an no screening			
Age 0-18 years			
Early cALD to 6 months after HSCT	1- rest[a]		
Early cALD to death	0.0741	1	Raymond et al. 2019 (9)
6 months after HSCT to >1 year after HSCT	1- rest[a]		1- rest[a]
6 months after HSCT to death	0.0741	1	Raymond et al. 2019 (9)
>1 year after HSCT to >1 year after HSCT	1- rest[a]		1- rest[a]
>1 year after HSCT to death	0.0003	1	Raymond et al. 2019 (9)
Late cALD to late cALD	1- rest[a]		Expert opinion
Late cALD to death	0.9000	3	Expert opinion

cALD cerebral ALD; HSCT Hematopoietic Stem Cell Transplantation; NBS newborn screening; ACTH Adrenocorticotropic Hormone;

[a]Rest means the rest of the transition probabilities of that health state

Supplemental 3: Results life years gained

Below a table of the deterministic results on life years gained.

Table S2: Results life years gained per case identified with ALD for the first 18 years

Analysis	Strategy	Costs	Life Years	Incr costs	Incr LY	ICER LY
Main analysis (Societal perspective)	Screened	€ 120,779	18.0	€ 57,865	1.9	€ 30,649
	Unscreened	€ 62,914	16.1			
Healthcare perspective	Screened	€ 104,311	18.0	€ 64,196	1.9	€ 33,787
	Unscreened	€ 40,115	16.1			
Utilities lower limit 95%CI	Screened	€ 120,779	18.0	€ 57,865	1.9	€ 30,649
	Unscreened	€ 62,914	16.1			
Utilities higher limit 95%CI	Screened	€ 120,779	18.0	€ 57,865	1.9	€ 30,649
	Unscreened	€ 62,914	16.1			
50% dies in three years from late cALD	Screened	€ 120,779	18.0	-€ 768	1.3	-€ 591
	Unscreened	€ 121,547	16.7			
60% dies in three years from late cALD	Screened	€ 120,779	18.0	€ 15,888	1.5	€ 10,592
	Unscreened	€ 104,892	16.5			
70% dies in three years from late cALD	Screened	€ 120,779	18.0	€ 30,731	1.6	€ 19,207
	Unscreened	€ 90,048	16.4			
80% dies in three years from late cALD	Screened	€ 120,779	18.0	€ 44,363	1.8	€ 24,646
	Unscreened	€ 76,416	16.2			
20% progressing to cALD	Screened	€ 96,208	18.0	€ 57,219	0.9	€ 65,626
	Unscreened	€ 38,988	17.1			
Screening costs without x-counter (tier 2)	Screened	€ 110,976	18.0	€ 48,062	1.9	€ 25,296
	Unscreened	€ 62,914	16.1			
Screening costs detecting five cases	Screened	€ 161,4093	18.0	€ 98,495	1.9	€ 51,840
	Unscreened	€ 62,914	16.1			

Costs are presented per patient. Incr incremental; LY life year; CI confidence interval; cALD cerebral ALD;

References

1. Huffnagel IC, Laheji FK, Aziz-Bose R, Tritos NA, Marino R, Linthorst GE, et al. The Natural History of Adrenal Insufficiency in X-Linked Adrenoleukodystrophy: An International Collaboration. J Clin Endocrinol Metab. 2019;104(1):118-26. doi: 10.1210/jc.2018-01307. PubMed PMID: 30252065.
2. Engelen M, van Ballegoij WJC, Mallack EJ, Van Haren KP, Kohler W, Salsano E, et al. International Recommendations for the Diagnosis and Management of Patients With Adrenoleukodystrophy: A Consensus-Based Approach. Neurology. 2022;99(21):940-51. Epub 20220929. doi: 10.1212/WNL.0000000000201374. PubMed PMID: 36175155; PubMed Central PMCID: PMC9687408.
3. Moser HW, Loes DJ, Melhem ER, Raymond GV, Bezman L, Cox CS, Lu SE. X-Linked adrenoleukodystrophy: overview and prognosis as a function of age and brain magnetic resonance imaging abnormality. A study involving 372 patients. Neuropediatrics. 2000;31(5):227-39. doi: 10.1055/s-2000-9236. PubMed PMID: 11204280.
4. Mallack EJ, Turk BR, Yan H, Price C, Demetres M, Moser AB, et al. MRI surveillance of boys with X-linked adrenoleukodystrophy identified by newborn screening: Meta-analysis and consensus guidelines. J Inherit Metab Dis. 2021;44(3):728-39. Epub 20210109. doi: 10.1002/jimd.12356. PubMed PMID: 33373467; PubMed Central PMCID: PMC8113077.
5. Gupta AO, Raymond G, Pierpont EI, Kemp S, McIvor RS, Rayannavar A, et al. Treatment of cerebral adrenoleukodystrophy: allogeneic transplantation and lentiviral gene therapy. Expert Opin Biol Ther. 2022;22(9):1151-62. Epub 20220919. doi: 10.1080/14712598.2022.2124857. PubMed PMID: 36107226.
6. Regelmann MO, Kamboj MK, Miller BS, Nakamoto JM, Sarafoglou K, Shah S, et al. Adrenoleukodystrophy: Guidance for Adrenal Surveillance in Males Identified by Newborn Screen. The Journal of Clinical Endocrinology & Metabolism. 2018;103(11):4324-31. doi: 10.1210/jc.2018-00920.
7. Moser HW, Smith KD, Watkins PA, Powers J, Moser AB. X-Linked Adrenoleukodystrophy. In: Valle DL, Antonarakis S, Ballabio A, Beaudet AL, Mitchell GA, editors. The Online Metabolic and Molecular Bases of Inherited Disease. New York, NY: McGraw-Hill Education; 2019.
8. Engelen M, Kemp S, de Visser M, van Geel BM, Wanders RJA, Aubourg P, Poll-The BT. X-linked adrenoleukodystrophy (X-ALD): clinical presentation and guidelines for diagnosis, follow-up and management. Orphanet Journal of Rare Diseases. 2012;7(1):51. doi: 10.1186/1750-1172-7-51.
9. Raymond GV, Aubourg P, Paker A, Escolar M, Fischer A, Blanche S, et al. Survival and Functional Outcomes in Boys with Cerebral Adrenoleukodystrophy with and without Hematopoietic Stem Cell Transplantation. Biol Blood Marrow Transplant. 2019;25(3):538-48. Epub 20181004. doi: 10.1016/j.bbmt.2018.09.036. PubMed PMID: 30292747.
